# From Presentation to Prognosis: Case-Based Learning in Pediatric Bacterial Meningitis for Postgraduate Training

**DOI:** 10.15766/mep_2374-8265.11632

**Published:** 2026-08-25

**Authors:** Geraldine Huynh, Jennifer Tam, Hilal Al Hashami, Manal Tadros, Anupma Wadhwa

**Affiliations:** 1 Clinical Assistant Professor, Division of Infectious Diseases, Department of Pediatrics, University of Alberta Faculty of Medicine and Dentistry; 2 Clinical Assistant Professor and Program Director, Division of Infectious Diseases, Department of Pediatrics, University of British Columbia Faculty of Medicine; 3 Clinical Consultant, Child Health Department, Royal Hospital; 4 Assistant Professor, Department of Laboratory Medicine and Pathobiology, Temerty Faculty of Medicine, University of Toronto; 5 Associate Professor, Division of Infectious Diseases, Department of Pediatrics, Temerty Faculty of Medicine, University of Toronto; 6 †Co-primary authors

**Keywords:** Case-Based Learning, Pediatrics, Infectious Diseases, Bacterial Meningitis, Microbiology

## Abstract

**Introduction:**

Pediatric bacterial meningitis is a life-threatening emergency with high morbidity and mortality. Missed diagnoses and treatment delays remain common, and teaching these skills can be challenging because the management requires learners to integrate complex clinical information and make time-sensitive decisions. We developed and evaluated a pediatric bacterial meningitis case-based learning (CBL) module to provide an interactive, evidence-based, and efficient teaching resource.

**Methods:**

This 90-minute module incorporates combined experience and expertise of 3 pediatric infectious diseases (PID) faculty, 1 PID trainee, and 1 microbiology faculty. Materials include a learner case with embedded questions, a facilitator guide, pre/post quizzes, and evaluations. We piloted the module in 2024 and evaluated the session with anonymous pre/post 10-item quizzes with pseudonyms used for a paired *t* test.

**Results:**

Seventy-five learners and 13 facilitators participated. Among 46 learner survey respondents, the overall session rating was 4.9/5; 100% reported that the learning objectives were met, and 93.5% endorsed an anticipated practice change. Forty-nine learners completed matched pre- and postquizzes. Mean quiz scores improved from 5.2/10 to 7.9/10, with a mean paired increase of 2.69 points (*SD* of paired differences 2.32; 95% CI, 2.03–3.36). This represented a statistically significant improvement by paired *t* test, *t*(48) = 8.13, *P* < .001. All facilitators reported reduced preparation time and would facilitate again.

**Discussion:**

This module was feasible to implement, highly rated, and improved learner knowledge with perceived behavioral impact. It represents the first open-access CBL resource focused on pediatric meningitis for postgraduate learners, addressing a key curricular gap.

## Educational Objectives

By the end of this activity, learners will be able to:

1. Apply an evidence-informed approach to the evaluation of infants and children with fever and altered mental status when bacterial meningitis is suspected.
2. Recognize the epidemiologic, microbiologic, and clinical features that should raise suspicion for bacterial meningitis in infants and children.
3. Identify indications and contraindications for lumbar puncture and interpret the effect of prior antibiotic therapy on the diagnostic yield of cerebrospinal fluid and blood cultures.
4. Select appropriate empiric antimicrobial therapy and adjunctive corticosteroid therapy for suspected bacterial meningitis and determine pathogen-directed treatment duration.
5. Recommend appropriate follow-up after bacterial meningitis, including evaluation for important long-term complications such as hearing loss.

## Introduction

Pediatric bacterial meningitis is a medical emergency associated with significant morbidity and mortality. Recent cohort and database studies continue to show important mortality and long-term morbidity after pediatric bacterial meningitis, including hearing loss, seizures, cognitive and developmental impairment, visual disturbances, and other neurologic disabilities.^1-6^ Prompt antimicrobial therapy is recommended when bacterial meningitis is suspected; adult community-acquired bacterial meningitis data associate delays beyond 2 hours with increased mortality.^7^ Despite this, studies show that one-third of infants receive inappropriate early treatment^8^ and nearly half of children with meningococcal infection were not sent to hospital after an initial visit.^9^ Given these risks, training pediatric residents to promptly recognize and manage meningitis in children is essential.

Teaching these skills can be challenging because the management of bacterial meningitis requires learners to integrate complex clinical information and make time-sensitive diagnostic and therapeutic decisions. Postgraduate learners and clinical supervisors must also balance formal education with competing patient-care responsibilities. Postgraduate curricula in postgraduate training programs often rely on didactic lectures, which are time consuming for faculty to prepare and less effective for learners. Organizers also struggle to recruit teachers to present. Traditional lecture-based approaches have been associated with lower examination performance and higher failure rates compared with active learning, highlighting the limitations of passive learning models.^10^ Case-based learning (CBL) is an active learning strategy that uses realistic clinical scenarios to help learners connect medical knowledge to clinical decision-making.^11,12^ CBL provides a more engaging, interactive, and effective experience. CBL modules support self-directed learning and adaptable teaching in various settings. While development requires effort, once created, CBL offers an effective, low-cost, and efficient tool.^13^

CBL can overcome the limitations of traditional teaching methods. In a systematic review of 104 papers that captured a variety of case types, timing, number, and length, students enjoyed CBL and found that it enhanced their training. Facilitators also valued CBL because it helped motivate students to come prepared and actively participate.^11^ In another systematic review, CBL was superior to other teaching methods, particularly didactic lectures and tutorial-based teaching, in terms of academic performance measured by exam scores, interest, and motivation.^14^

Although resources such as *Challenging Cases in Pediatric Infectious Diseases*^15^ provide breadth, they lack the depth needed for postgraduate teaching. To our knowledge, open-access offerings specific to postgraduate learners in pediatric infections diseases (PID) that provide in-depth teaching on a topic and a flexible format adaptable to multiple stages of postgraduate learning are limited. We developed this pediatric bacterial meningitis CBL module to provide an interactive resource that can be adapted to various levels of postgraduate learners.

## Methods

### Development of the Resources

This resource applies knowledge and clinical reasoning to pediatric bacterial meningitis. We created the module between 2021 and 2024. The development team included 3 PID faculty, 1 PID trainee, and 1 microbiology faculty at 2 Canadian quaternary centers. To enhance face validity, the case was derived from a composite of multiple clinical encounters. The facilitator guide (Appendix A) and trainee guide (Appendix B) were written with reference to Canadian Paediatric Society guidance, *Nelson Textbook of Pediatrics*, peer-reviewed studies, and local antibiograms.^16,17^

Thirty interspersed questions were crafted to promote reasoning, knowledge application, and critical thinking, with difficulty levels that facilitators can tailor to learner experience. Topics without consensus, such as dexamethasone use, were intentionally included to mirror real-world complexity. Materials underwent iterative review and piloting in self-directed, one-on-one, and small-group formats. Feedback from learners and faculty informed revisions to ensure clarity, flow, and educational value. Based on that feedback, a “how-to” instruction PowerPoint for the manual was included (Appendix C). Further feedback can be elicited with the surveys for both participants and facilitators (Appendices D and E).

The trainee feedback form captured site, level of training, preparation for the session, perceived usefulness of presession materials, facilitator preparedness, engagement, active learning, achievement of learning objectives, and realism and level of challenge of the case, as well as open-ended reflections on new learning, impact on practice, and willingness to participate again. Facilitators completed a separate postsession form capturing site, learner audience, number of learners, delivery mode, preparation activities and time, estimated time saved compared with preparing their own teaching materials, feedback on the case, answer key and articles, observed impact of the case-based format on clinical reasoning and critical thinking, and willingness to facilitate a similar session again.

This project was reviewed in consultation with the SickKids Research Ethics Office and the University of British Columbia (UBC) Research Ethics Board (REB), who confirmed that it constitutes a medical education activity rather than human subjects research requiring REB review; furthermore, as the surveys were fully anonymous, formal ethics approval was waived.

### Curricular Context

We implemented the refined version of our case-based module in July 2024 as part of the pediatric residency program academic half-day curriculum. Each small group included approximately 8 residents and 1 facilitator. Facilitators included PID attending physicians or PID subspecialty fellows. Sessions lasted approximately 90 minutes.

While evaluated in a small-group academic half-day format, the module is adaptable for one-on-one clinical teaching or independent self-directed learning, using the answer key for guidance. We involved PID attendings and subspecialty fellows in our evaluation; however, facilitators can include pediatricians or other advanced trainees with prior clinical experience in this topic.

### Implementation of the Activity

Approximately 1.5 weeks prior to the scheduled session, learners completed a “pretest” of 7 multiple-choice questions found at the end of Appendix B, Appendix F, and Appendix G. Answers are found in Appendix G. For the purposes of evaluation, we asked trainees to label their pretest with a pseudonym they could remember so that we could match posttest results. About 1 week prior to the scheduled session, learners received the case (see Appendix B) in advance with suggested readings. Learners were encouraged to go through the questions themselves first.

For the small-group session, a 90- to 120-minute session was booked at each institution during the pediatric residents’ academic half-day, with space and facilitators to support a maximum of 8 learners per group. Our sessions were held in person, although virtual and hybrid sessions are possible and have also been conducted using the same resource.

At the session, facilitators guided discussion through the case using Appendix A for support, which includes answers and contextual explanations to deepen learner knowledge. The discussions focused on outstanding questions that learners had after going through the case themselves in advance, allowing for high-yield discussion with the facilitator. At the end, learners completed a 10-question posttest quiz included at the end of Appendix B, labeled with their pretest pseudonym.

Session length and discussion depth can be tailored to the learner's experience and familiarity with the topic. Appendix C provides guidance for facilitators and learners on adapting the module across training levels and contexts. Questions may be emphasized or skipped based on time or learning needs: for example, questions 1-7 focus on evaluating fever and altered mental status in infants, questions 8-25 on treatment in infants and older children, and questions 26-30 on complications and prognosis. Senior learners familiar with initial assessments may focus on questions 8-18 and 24 to review key management points and delve deeper into areas of controversy and emerging evidence.

### Evaluation of the Activity

Learners completed a session evaluation (see Appendix D) at the end of the activity reflecting on their learning, anticipated practice changes, and session quality. This included both quantitative and qualitative questions typical of postsession evaluation forms for quality improvement. Facilitators completed an evaluation at the end of the session (see Appendix E), addressing case quality, feasibility, and teaching experience. This included both quantitative and qualitative questions typical of postsession evaluation forms for quality improvement.

We also evaluated the activity by assessing knowledge acquisition by comparing learners’ pre- and posttest results (see Appendices F and G) using a paired Student *t* test to determine whether the change in learners’ scores after the intervention was statistically significant. For the purposes of our module evaluation, pre- and posttest results were linked only by pseudonyms chosen by trainees.

### Validity Evidence

We sought evidence across 6 domains**.** For content validity, experts in PID and microbiology reviewed all materials for alignment with guidelines, literature, and clinical practice. Topics without consensus, such as steroid use, were intentionally included to reflect real-world complexity.^16,17^ For response process validity, we piloted the module in informal one-on-one and small-group formats and revised questions for clarity. We then launched the module in a formal small-group academic half-day session and adapted the materials based on formal and informal feedback. We created a standardized facilitator guide and slide deck to ensure consistent delivery. Learners used pseudonyms on pre- and posttests to reduce bias and allow paired analysis. For internal structure validity, we embedded pre/post quizzes to measure knowledge change, with paired analysis planned to evaluate whether there was a statistically significant improvement. In regard to the relationship to other variables validity, quiz items were aligned with CPS guidance and *Nelson Textbook of Pediatrics* to support construct validity.^16,17^ For consequences validity, the module was designed as a formative, low-stakes assessment. Evaluation forms captured impact on knowledge, reasoning, anticipated practice changes, and teaching efficiency. Its freely accessible, resource-light design promotes equitable dissemination across diverse settings. Lastly, for face validity, this was assessed through learner and facilitator feedback on the perceived relevance and clinical applicability of the case.

## Results

### Participants

We delivered the module to 75 learners across 13 small-group sessions at 2 large pediatric residency programs. The University of Toronto core pediatric residency program has approximately 100 residents, and the UBC pediatric residency program has approximately 80 residents across 3 sites.^18,19^ Of the 46 postmodule responses (61.3% response rate), 36 (78.3%) were from first-year residents, 6 (13.0%) were from second-year residents, 2 (4.3%) were from third-year residents, and 2 (4.3%) were from medical students. There were no fourth-year residents, as they attended a separate academic half-day. Thirteen facilitators (5 faculty, 8 fellows) led the groups.

### Knowledge Outcomes

Seventy-five learners and 13 facilitators participated. Among 46 learner survey respondents with a response rate of 61.3%, the overall session rating was 4.9/5; 100% reported that the learning objectives were met, and 93.5% endorsed an anticipated practice change. Forty-nine learners completed matched pre- and postquizzes. Mean quiz scores improved from 5.2/10 to 7.9/10, with a mean paired increase of 2.69 points (*SD* of paired differences 2.32; 95% CI, 2.03–3.36). This represented a statistically significant improvement by paired *t* test, *t*(48) = 8.13, *P* < .001. Because of anonymization, we could not link quiz performance with level of participation or observations from the facilitator on clinical reasoning during the session. While the quiz primarily assessed interpretation and treatment, broader objectives, such as counseling families and long-term management, were addressed qualitatively in discussion.

### Learner Feedback

We received 46 learner evaluations (18 UBC, 28 Toronto) with a response rate of 61.3%. Learners globally rated the session 4.9/5, with high scores for challenge (4.7/5), realism (4.9/5), engagement (5/5), and achievement of objectives (4.9/5).

Free-text comments were reviewed descriptively. Learners commonly described the cases as engaging, clinically relevant, and helpful for applying knowledge to management decisions. Representative comments included:

• “Thought this was a phenomenal session. Information stuck much better due to case-based nature.”
• “This approach is more realistic and closer to daily practice.”
• “Great consolidation of knowledge and reasoning.”

Overall, 93.5% reported that the session would change their clinical practice, including ensuring hearing screening, using molecular diagnostics when appropriate, and tailoring empiric antibiotics by age.

### Facilitator Feedback

There were 13 facilitators, and feedback was obtained from 10 of them with a response rate of 76.9%. To prepare, all facilitators read the provided answer key. Two facilitators chose to read some of the additional suggested readings. Five facilitators reviewed personal notes prior to the session, and 1 facilitator reviewed previous talks on this topic. The amount of time spent preparing to facilitate the session ranged from 30 to 180 minutes. Facilitators stated that they would normally require, on average, at least 6 hours to prepare a didactic talk for an academic curriculum session if they had to prepare materials on their own; therefore, preparation time was reduced for all facilitators. All facilitators noted that the resources shared ahead of time were helpful and that the format promoted interest, participation, and engagement. Comments from facilitators included:

• “Stimulated both practical and theoretical questions that benefitted the entire group.”
• “Learners were more engaged than in lectures; discussion was tailored to their needs.”

### Validity Evidence

Validity evidence was sought across 6 domains. Content validity was supported through expert review by PID and microbiology faculty in alignment with CPS guidance, *Nelson Textbook of Pediatrics*, and clinical practice.^16,17^ Response process validity was supported by piloting the session, revising unclear questions, using standardized facilitator guides and slides, and using pseudonyms for pre/posttests. Internal structure validity was demonstrated by significant improvements in learners’ pre/postquiz score gains. Evidence related to relationship to other variables was supported by the alignment of learner performance and discussion with the constructs emphasized in CPS guidance and *Nelson Textbook of Pediatrics*.^16,17^ Consequences validity was supported by learners’ reported anticipated practice changes, as well as facilitators’ reported reduced preparation time compared to creating de novo content, supporting both educational impact and teaching efficiency. Finally, face validity was supported by learner and facilitator feedback indicating that the module was realistic and clinically relevant and reflective of the common challenges encountered in the diagnosis and management of pediatric bacterial meningitis.

## Discussion

This pediatric bacterial meningitis CBL module was feasible to implement, highly rated, and demonstrated measurable knowledge gains across 2 institutions. Learners valued the interactive, authentic, and clinically relevant format. Facilitators reported reduced preparation burden and greater engagement.

Multiple sources of validity evidence supported the module. For example, the module was based on real cases to allow for high face validity. Writing of the case and answer key was done rigorously, with appropriate evidence-based references and input from experts in infectious diseases and microbiology. In addition, we included areas without consensus in the literature, such as the use of steroids, to promote in-depth discussion and understanding of nuanced issues in clinical care. This module has also been used one-on-one and as a self-directed learning resource. While not formally evaluated in these settings, informal feedback has been positive. We anticipate that it will continue to be helpful in several teaching settings.

Using Miller's pyramid, the module primarily assessed Knows and Knows How through the pre/postquiz and application of knowledge to case-based questions.^20^ Although facilitated discussions allowed learners to work through clinical reasoning as a group, individual performance was not directly observed through simulation, role-play, or workplace-based assessment. While we did not directly assess the Shows How or Does level, most learners reported anticipated practice change, suggesting transfer to clinical care.^20^ This module also aligns with the Canadian Royal College of Physicians and Surgeons of Canada Pediatrics Foundations Entrustable Professional Activity (EPA) 1 (“Recognizing deteriorating and/or critically ill patients and initiating stabilization and management”) and Pediatrics Core EPA 11 (“Coordinating transitions of care for patients with medical or psychosocial complexity”),^21^ and with the Association of American Medical Colleges Core EPA #10 (Urgent/emergent care) for graduating medical students.^22^

Viewed through the lens of Miller,^20^ our evaluation confirmed that learning occurred: paired quizzes showed statistically significant improvement, and 93.5% of learners reported anticipated changes in practice. Numerous learner comments noted enjoyment of the case, and all facilitator responses noted that they also enjoyed CBL. While enjoyment alone does not prove learning, these findings demonstrate that engagement was coupled with measurable gains. The pre/posttest primarily focused on the interpretation of microbiologic tests, primarily cerebrospinal fluid, and treatment; broader objectives such as counseling families and long-term prognosis were addressed through discussion but not directly assessed.

This module is, to our knowledge, the first systematically developed, evidence-based CBL resource on pediatric bacterial meningitis for in-depth postgraduate training. It is adaptable across learner levels and instructional formats, addressing a critical curriculum gap in PID education. The module incorporates multiple domains of validity evidence and is explicitly aligned with Miller's pyramid, as well as national and international EPAs, supporting its rigor and transferability across training programs. By providing a flexible, time-efficient, and openly accessible resource, this module supports high-quality postgraduate teaching in diverse clinical and educational settings.^20-22^

### Strengths and Limitations

Strengths include expert-informed development, grounding in real cases, structured and referenced facilitator resources, and evaluation within a real-world setting, enhancing external validity. Limitations of the resource include the time that it may take to organize the space and facilitators required for this format. Other limitations may include dependence on facilitator skill, narrower scope with emphasis on depth over breadth, and variable discussion quality between groups. Generalizability may be limited by piloting at 2 centers and by the resource being available only in English, though the resource-light design supports broader adoption in high- and low-resource settings.

Limitations in the evaluation of this module include reliance on a pre/postdesign without a control group, potential test-retest effects, inability to measure long-term retention, and restricted quiz content coverage. We also did not directly compare faculty preparation time for this CBL module with preparation time for an existing lecture on the same topic. Therefore, while the module may reduce the need for de novo content development, we cannot conclude that it saves faculty time compared with reviewing or delivering established lecture materials. The lower posttest completion rate represents a limitation of this study. Because reasons for participant dropout were not collected, we cannot determine whether learners who completed the posttest differed systematically from those who did not, potentially introducing response bias.

Future directions include developing additional modules on other PID topics to form a comprehensive, high-quality, and openly accessible PID Casebook; conducting further scholarly evaluation to assess long-term knowledge retention and correlations with in-training exam performance; and refining virtual and hybrid delivery formats to support interactive, scalable dissemination across diverse educational settings.

### Conclusion

Pediatric bacterial meningitis is a life-threatening emergency that requires rapid diagnosis and treatment. Preparing pediatric postgraduate trainees to promptly recognize and manage this fatal condition is therefore essential. Traditional didactic teaching, however, is time intensive for faculty and less engaging for learners, highlighting the need for interactive, evidence-based resources.

We developed a CBL module on pediatric bacterial meningitis that improved learner knowledge, was highly rated, and reduced faculty preparation time compared to creating their own content de novo. To our knowledge, it is the first open-access, evidence-based resource on this topic, explicitly aligned with competency frameworks. By incorporating multiple domains of validity evidence and aligning with Miller's pyramid and national and international EPAs, this module addresses a critical curriculum gap in PID and is ready for dissemination across training programs.^20-22^

## Appendices


Module - Facilitator Version.docxModule - Student Version.docxHow to Use Manual.pptxTrainee Feedback Form.docxFacilitator Feedback Form.docxPre- and Posttest - Blank.docxPre- and Posttest - Answers.docx
*All appendices are peer reviewed as integral parts of the Original Publication.*
