# Supplementary material for: From Presentation to Prognosis: Case-Based Learning in Pediatric Bacterial Meningitis for Postgraduate Training
Source: MedEdPORTAL. 2026 Aug 25;22:11632. doi: 10.15766/mep_2374-8265.11632 (PMC13503623; doi:10.15766/mep_2374-8265.11632)
Supplement: Supplementary file 1 [file mep_2374-8265.11632-s001.zip › A. Module - Facilitator Version.docx]

**An Infant with Fever and Altered Mental Status**

Case-based learning session

**The Case:**
Oscar is an 8-month-old boy whom you are seeing in the Emergency Department. His mother reports that he has had a runny nose and fever for the past few days. She gave him acetaminophen and a dose of amoxicillin that she had left over from an illness one month prior, which seemed to help his fever. Today, however, she noticed that he was not interested in eating or drinking and was acting very sleepy. Worried, she brought him to the hospital.

**Past Medical History:**

- Born at term via spontaneous vaginal delivery, no perinatal or neonatal complications
- No previous hospitalizations or surgeries
- Treated for an ear infection one month ago
- Immunizations are up to date for age, as per the local immunization schedule for his age with adequate doses of pneumococcal conjugate vaccine for age

**Social History:**

- Attends day care 5 days a week
- Lives with his parents and two siblings (4-year-old who attends daycare; 7-year-old is in Grade 1)

**On Physical Exam:**

- General appearance:	Lethargic and ill-looking with mottled skin, capillary refill 3-4 seconds
- Vitals:	T 39.7°C, HR 156 bpm, RR 37 breaths/min, BP 85/48 mmHg, SpO2 98%
- Weight:	8.9 kg
- HEENT:	Erythematous left tympanic membrane, normal positioned pinnae, no tenderness to palpation over the mastoids
- NEURO:	Irritable cry in response to pain, eye opening to verbal stimuli, limbs withdraw to touch, flat anterior fontanelle, pupils equal and reactive to light, possible increased crying on passive flexion of the neck, Kernig and Brudzinski signs negative
- Chest:	Clear, no increased work of breathing
- CVS:	Sinus tachycardia, normal heart sounds S1 & S2, no murmur
- Abdomen:	Soft, non-tender, bowel sounds present

1) **Summarize the pertinent points of the case so far.**

8-month-old boy with fever and altered mental status after a few days of URTI symptoms. On exam, he has evidence of otitis media and possibly neck stiffness. He is fully immunized for age (including conjugate pneumococcal vaccine). He is currently taking amoxicillin. He has been treated with antibiotics for otitis media in the last month.

2) **What is the differential diagnosis you should consider?**

Meningitis

Sepsis

Encephalitis

Brain abscess or parameningeal abscess

Mastoiditis?

Less likely: malignancy, cerebral vasculitis, toxin exposure

3) **What are the Kernig’s and Brudzinski’s signs? How sensitive are these signs in children?**

If positive or present, these signs suggest meningeal and nerve root inflammation. These signs are the result of physical maneuvers as described here:

Kernig’s sign:	Positive when patient is lying supine, hip is flexed 90 degrees, and there is subsequent pain on extension at the knee

Brudzinski’s sign:	Positive when there is involuntary flexion of the knees and hips after passive flexion of the neck when lying supine

Not often positive in children less than 12-18 months of age.^1^
A 2011 prospective study of children age 2 months to 16 years old in Israel (n=108) showed^2^:

|  | Sensitivity | Specificity |
| --- | --- | --- |
| Kernig’s Sign | 27% | 87% |
| Brudzinski’s Sign | 51% | 80% |

Although Brudzinski’s original article cited a sensitivity of 96% for Brudzinski’s sign and 57% of Kernig’s sign, more recent studies on adult bacterial meningitis have reported a sensitivity of 5% for both Brudzinski’s and Kernig’s signs.^3^

4) **What is Oscar’s Glasgow Coma Scale score?**

11/15

| Eyes | Opens to verbal stimuli | 3 |
| --- | --- | --- |
| Verbal | Cries in response to pain - “consolable crying” | 4 |
| Motor | Withdraws | 4 |

**GLASGOW COMA SCALE**

| **Eye opening (total possible points 4)** | | | |
| --- | --- | --- | --- |
| Spontaneous | 4 | | |
| To voice | 3 | | |
| To pain | 2 | | |
| None | 1 | | |
| **Verbal Response (Total possible points 5)** | | | |
| ***Older Children*** | | ***Infants and Young Children*** | |
| Oriented | 5 | Appropriate words; smiles, fixes and follows | 5 |
| Confused | 4 | Consolable crying | 4 |
| Inappropriate | 3 | Persistently irritable | 3 |
| Incomprehensible | 2 | Restless, agitated | 2 |
| None | 1 | None | 1 |
| **Motor Response (Total possible points of 6)** | | | |
| Obeys | 6 | | |
| Localizes pain | 5 | | |
| Withdraws | 4 | | |
| Flexion | 3 | | |
| Extension | 2 | | |
| None | 1 | | |

Ref: (4)

5) **Does Oscar have evidence of increased intracranial pressure (ICP)? Why or why not?**

Maybe: altered mental status (but not stuporous, or coma)

Reassuring signs suggesting he does not have increased ICP: flat fontanelle, pupils equal and reactive to light (no anisocoria), no hypertension with bradycardia, no apnea or hyperventilation, no history of vomiting or obvious headache (but he is 8 months, so it may be hard to tell if he has a headache), no abnormal posturing

Overall, elevated ICP does not seem likely, but important to consider in any patient with altered mental status/lethargy.

6) **What further information on history and/ or physical exam would be helpful in your assessment?**

Several possible answers here. As the learner asks for more information, the facilitator can ask what they are thinking, and further probe how this information would be helpful.

Examples:

- Papilledema, CN III or VI palsy (signs of ICP)
- Focal neurological findings (signs of vascular occlusion)
- Kernig’s, Brudzinski’s, stiff neck, back pain (signs of meningeal irritation)
- Tuberculosis (TB) risk factors on history
- Petechial rash (especially with meningococcal sepsis)
- History of sick contact
- Travel history
- Animal contact

7) **What initial management and work-up would you do?**
1. ABC’s, monitoring, consider 1:1 nursing (given altered mental status)
2. Start an IV with bloodwork, including: CBC with differential, electrolytes, creatinine, urea, liver enzymes, blood gas, urinalysis and urine culture, **blood culture**
3. Lumbar puncture (LP): cell count, protein, glucose, Gram stain, bacterial culture; consider HSV PCR, acid fast bacilli smear and mycobacterial culture, and others depending on risk factors; if concern for elevated ICP, consider CT head first (but seems less likely in this case)
4. Begin IV fluids and order antibiotics – if LP will delay antibiotics, give antibiotics first.
8) **A medical student asks you: “How useful is doing a blood culture when you are clinically suspecting meningitis?” How do you reply?**

Blood cultures are positive in up to 80-90% of cases of meningitis prior to antibiotics, so should be performed in all patients with suspected meningitis.^1^ (Oscar has been taking amoxicillin, but still may be helpful if an organism grows.)

You are planning to do a lumbar puncture (LP) as part of your initial work-up.

9) **Name 3 contraindications to consider before doing your LP.**
1. Evidence of increased ICP (other than bulging fontanelle) or signs of herniation
   Examples: 3^rd^ or 6^th^ nerve cranial nerve palsy with depressed level of consciousness, hypertension with bradycardia, and respiratory abnormalities
2. Unstable clinical status such as shock, or in patients in whom positioning for LP would further compromise cardiopulmonary function
3. Skin infection overlying site of the LP
4. Coagulopathy (thrombocytopenia is a relative contraindication)

Ref: (1,5)

10) **If you suspect increased ICP, what should be done prior to attempting an LP?**

CT head with contrast and/or MRI brain

Manage ICP prior to attempting an LP (if cannot be managed, do not do LP)
Head imaging should not delay antibiotic therapy

Ref: (1,5)

11) **Suppose performing an LP was delayed by several days due to contraindications, but now, those are no longer present and an LP could be performed. What useful information could an LP provide despite the patient already having received antibiotics for a few days? What additional test you may consider in the CSF to increase the yield of knowing the causative organism?**

Even if already on antibiotics, the **cell count & chemistry** will be helpful as pleocytosis with a neutrophil predominance, elevated protein, and low glucose may persist for several days despite antibiotics^.^ Therefore, an abnormal cell count & chemistry profile may be helpful, although a normal cell count & chemistry panel may not necessarily rule meningitis out.

Ref: (1)

Molecular testing could also be performed on the CSF sample. Since these tests detect nucleic acid, they do not require viable organism to grow and therefore may be able to detect the presence of bacterial nucleic acid remnant material even after killing from antibiotics has occurred. Examples of molecular tests include: specific targeted bacterial PCRs (which may be individually performed or part of a panel), or **16s ribosomal RNA testing**. If a sequence is detected, it is compared to a database of known bacteria in order to identify the suspected bacteria. Note that specific bacterial PCRs are preferred over 16s because bacterial PCRs are more sensitive.

The specific molecular testing available for CSF varies between centers. Becoming familiar with what is locally available to you (which are the preferred tests, what organisms does it look for, and what is the sensitivity and specificity?) is important. It is also important to note that negative molecular testing would not rule out meningitis.

12) **How often is the CSF culture positive in community acquired bacterial meningitis if no previous antibiotics have been given? What about if antibiotics have been started?**

    Pediatric studies have shown sterility of 90-100% of CSF cultures within 24-36h of appropriate antibiotic treatment, with more recent studies suggesting even more rapid sterility (2 hours in cases of *Neisseria meningitides* (meningococcus), 4 hours in cases of *Streptococcus pneumoniae* (pneumococcus)). Molecular/PCR tests may be helpful in these cases. After antibiotic initiation, CSF culture yield decreases rapidly and is time-dependent rather than a single fixed percentage. Reported culture positivity decreases from approximately 85% before antibiotics to 73% when CSF is obtained within 4 hours of antibiotic administration, 11% when obtained 4-8 hours after antibiotics, and 0% when obtained more than 8 hours after antibiotics. Gram stain may remain positive after antibiotic exposure, although its yield is reduced. Therefore, when lumbar puncture is delayed until after antibiotics have been started, negative CSF culture does not exclude bacterial meningitis, and molecular/PCR testing may be helpful.

Ref: (5, 14. 15)

You would like to begin antibiotics even before test results return because meningitis and sepsis are on your differential and you do not want to delay treatment.

13) **What organisms do you want to cover?**

|  | **Bacteria** |
| --- | --- |
| **Neonate (0-31 days)** | *Group B Streptococcus (Streptococcus agalactiae)*  *Escherichia coli*  *Listeria monocytogenes* |
| **Infant (1 months-3 months)** | *Group B Streptococcus (Streptococcus agalactiae)*  *Escherichia coli*  *Listeria monocytogenes*  *Streptococcus pneumoniae*  *Neisseria meningitidis*  *Haemophilus influenzae* |
| **Child (3 months – 18 years)** | *Streptococcus pneumoniae*  *Neisseria meningitidis*  *Haemophilus influenzae* |

14) **Which antibiotics would you start and why?**

**Cefotaxime/ceftriaxone** at meningitic doses and some centres will also start **vancomycin**

**Teaching point:** Vancomycin is for the possibility of a cefotaxime/ceftriaxone-resistant *S. pneumoniae* (not for MRSA in this case as this would be an uncommon cause of meningitis).The rates of resistance will need to be compared to a local antibiogram if available. However surveillance studies from 2020 showed that 90.1% and 98.6% of invasive pneumococci were penicillin-susceptible when MICs were interpreted using The Clinical & Laboratory Standards Institute (**CLSI**) meningitis or oral and non-meningitis breakpoints, respectively; 96.9% (meningitis breakpoint) and 99.5% (non-meningitis breakpoint) of isolates were ceftriaxone-susceptible in Canada (6).

In areas where there have not been cephalosporin-resistant *S. pneumoniae* cases, a 3rd generation cephalosporin alone (without the addition of vancomycin) may be adequate empiric therapy. However, pending culture results, most experts recommend adding vancomycin to the third-generation cephalosporin to protect against the possibility of a cephalosporin-resistant *S. pneumoniae*, which has emerged in some parts of Canada and the USA.

Ref: (6, 10)

15) **What if the patient was allergic to B-lactam antibiotics?**

For penicillin allergy:	vancomycin + ceftriaxone

For ceftriaxone allergy/intolerance:	Meropenem

Ceftriaxone/cefotaxime doesn't share a side-chain with penicillin/amoxicillin, so for type I IgE mediated reactions, ceftriaxone/cefotaxime can be used.

Ref*:* (11)

16) **Would you start adjunctive dexamethasone? Why or why not?**

The rapid killing of bacteria in the CSF can precipitate a cytokine-mediated inflammatory cascade. The resultant edema may lead to further neurologic injury, so theoretically corticosteroids when administered in the correct time frame may mitigate this damage.

In children, dexamethasone given just before or within 2h of the first antibiotic dose decreased hearing loss in *H. influenzae* type b meningitis^7^. The evidence for *S. pneumoniae* meningitis is less conclusive in pediatrics, but has been shown to have improved outcomes (including decreased mortality) in adults before or with the first dose of parenteral antibiotics^8^. It has not been shown to be helpful after CNS damage has already occurred.

The dosing for dexamethasone for this indication is 0.6 mg/kg/day IV divided q6h x 2-4 days given within 2 hours^7^. This is specifically for *H. influenzae type b* or *S. pneumoniae meningitis.* If a different etiology is identified, steroids can be discontinued, as there is no evidence of benefit in continuing steroids for other causes. Of note, rebound fever may occur after steroids are discontinued.

A key concern with dexamethasone is that by reducing inflammation, it may limit antibiotic penetration into the CSF. In developing countries, it has been less effective in preventing complications like hearing loss, possibly due to factors like HIV or advanced disease. Animal studies suggested that dexamethasone might reduce vancomycin penetration in cases of resistant *S. pneumoniae*, but this was not confirmed in studies with human children.^8, 9^

Overall, the evidence suggests a benefit of a short course of steroids given before or within 2h of initial antimicrobial therapy in acute meningitis caused by either *H. influenzae b* or *S. pneumoniae*.

Ref: (1,5, 7, 8, 9)

The following are select preliminary lab results:

**CBC**:	Hgb 154 g/L, RBC 5 x 10^12^/L, Platelets 297 x 10^9^/L, WBC 21 x 10^9^/L, neutrophils 40%, bands 24%, lymphocytes 34%

**Renal function & liver enzymes**: Normal

**Urinalysis**: Normal

**Urine culture:** Pending

**Blood culture:** Pending

**CSF**: Xanthochromic, WBC 210 x 10^6^/L (70% neutrophils), RBC 2820 x10^6^/L, glucose 0.2 mmol/L, protein 2.8 g/L; culture pending

17) **Fill in the following table with ++/ +/ normal / - / -- to describe typical cell count and chemistry from CSF in the various infectious meningitic conditions:**

| **Component** | **Abnormal Values** | | |
| --- | --- | --- | --- |
|  | **Bacterial** | **Viral** | **TB** |
| **WBC (x10^6/L)** | **++**  100-10,000 (++) | N/**+**  Rarely >1000 | N/**+**  10-500 |
| **%neutrophils** | **+**  (75-95) | N/**+**  Neutrophils early on; lymphocytes and monocytes predominate later | N/**+**  Neutrophils early on; lymphocytes predominate later |
| **Protein (g/L)** | **+**  1-5 | N 0.5-2 | **++**  1-30 |
| **Glucose (mmol/L) (% of serum)** | **--**  <2.2  <4.0 (<6.0 in neonates) | N | **-**  <5.0 |

N=normal; absolute numbers are for facilitator’s info(1)

Note that ~10% of acute bacterial meningitis can have lymphocyte predominance initially (especially neonatal Gram-negative bacillary meningitis and *Listeria monocytogenes* meningitis). A 2016 study in children (n=175) showed that 16% of children (aged 1 month to 18 years old) with culture-proven bacterial meningitis had low CSF WBC (0-20/mm^3^) . Having a low CSF WBC counts despite high CSF bacterial counts can be a sign of poor prognosis.^12^

18) **How would you interpret the cell count of a traumatic LP?**
    Bottom line: you can’t.
    A number of different methods for “correcting” the WBC count based on RBC count have been developed, but none are reliable and can be falsely reassuring.

Two commonly used methods for adjustment:
1. **“1:500” ratio.** *This is a generally more conservative ratio to minimize falsely reassuring results; some clinicians may use even more conservative ratios such as 1:1000 (ref CPS statement on fever <90d old).* Using the assumption that the ratio of WBC to RBC in blood is 1 in 500, one can deduct 1 WBC for every 500 RBC in the CSF
E.g., in this case, with 2820 RBC, one can deduct 2820/500 = 5.64 = ~6 WBC. This leaves 210 – 6 = 204 WBC - still high!
2. **WBC/RBC ratio based on peripheral blood CBC**
WBC_CSF_/RBC_CSF_= WBC_blood_/RBC_blood_
Predicted WBC_CSF_ = RBC_CSF_ x (WBC_blood_/RBC_blood_) ****Make sure units are the same, especially as SI units for WBC_blood_ is 10^9^/L and RBC_blood_ is 10^12^/L ; also make sure to use RBC and not Hgb****

Adjusted WBC_CSF_ = Actual WBC_CSF_ – Predicted WBC_CSF_

E.g., in this case, Predicted WBC_CSF_ = 2820 x (21/5000) = 11.8. Adjusted WBC_CSF_ = 210 – 11.8 = 198.2 (still high!)

In a traumatic tap, results from the gram stain and culture can still be helpful. The CSF protein will be elevated due to RBCs.

Ref: (13)

Oscar’s CSF Gram stain shows Gram positive cocci in pairs (CDC):

Centers for Disease Control and Prevention (CDC). Image ID 18081 [image]. Public domain. Atlanta (GA): CDC; [cited 2026 Feb 4]. Available from: <https://wwwn.cdc.gov/phil/Details.aspx?pid=18081>

19) **What is your working diagnosis now?**
    Bacterial meningitis with likely *S. pneumoniae* based on the Gram stain.
    *Reminder*: do not use Gram stain results to narrow therapy (would await for culture confirmation), but may be useful to broaden therapy if it suggests an etiology not currently being covered by antibiotics.
20) **List risk factors for bacterial meningitis.**
    Demographics
    1. Young age (lack of immunity to specific pathogens)
    2. Black or Native American race – may be more related to low SES (note that H. influenzae type A more common in Indigenous communities)
    3. Male sex

Bacteria present

4. Recent colonization with pathogenic bacteria
5. Infection with causative organism (e.g., otitis media, pneumonia, occult bacteremia, sinusitis)
6. Occult bacteremia (e.g. with meningococcus, *H. influenzae* type b)

Exposures

7. Close contact (household, daycare, college dorm, military barracks) with individual(s) with invasive disease caused by *N. meningitis* or *Hib*
8. Crowding
9. Poverty

Immune system deficits

10. Splenic dysfunction (including Sickle Cell Disease): encapsulated pathogens – *Hib, S. pneumoniae, N. meningitidis*
11. Complement (C5-8)/properdin (alternative complement pathway) defects: *N.* meningitidis
12. Acquired or congenital T lymphocyte defects (e.g. AIDS, steroids, chemotherapy): L. monocytogenes
13. Incompletely or non-vaccinated individuals (S. pneumoniae, Hib, N. meningitidis) – remember that it takes about 2 weeks for the vaccine to be effective
14. Prematurity

Anatomical defects

15. Congenital or acquired CSF leak across a mucocutaneous barrier
16. Cochlear implants: *S. pneumoniae*
17. Lumbosacral dermal sinus and meningomyelocele: *S aureus,* Gram negative enterics

Ref: (16, 17, 18, 19)

21) **What is the likely mode of transmission in meningitis?**
    Person-to-person transmission by respiratory tract secretions or droplets.
22) **How do bacteria get to the meninges? What was the most likely route in Oscar?**

Most often through **hematogeneous spread** from a distant site of infection (or nasal carriage), but can also be from **direct bacterial invasion from a contiguous site of infection** (otitis media, sinusitis, vertebral osteomyelitis, mastoiditis).

For Oscar, while he did have otitis media earlier and contiguous spread from this is possible, he did not present with signs of mastoiditis or complicated otitis media. Therefore, it is most likely that the route of infection in his case was hematogenous.

23) **List signs and symptoms of meningitis in infants and in older children.**

| Infants | Older Children |
| --- | --- |
| - Typically non-specific - Fever, poor feeding, vomiting, inconsolable crying, lethargy - Bulging fontanelle, diminished activity, septic appearance, petechial rash | - Fever, vomiting, back/neck pain, confusion/disorientation, photophobia - Neck stiffness, Kernig’s sign, Brudzinski’s sign, petechial rash, focal neurological findings |

After 48 hours, Oscar’s status is improving. He is more interactive with his parents and his fever trend is improving. His CSF culture was negative, but his blood culture grew *S. pneumoniae*, which was **intermediately susceptible to penicillin** (MIC 0.06-0.12 mcg/mL) and **susceptible to cefotaxime** according to CNS breakpoints.

24) **What antibiotic would you use to treat Oscar? How long do you anticipate the duration of treatment would be (assuming he does not develop any complications)?**

Cefotaxime or ceftriaxone (meningitic doses) for 10-14 days, with the entire course parenterally (IV/IM).

**Assuming uncomplicated meningitis, what duration would you use if the causative organism had been each of the following::**

***N. meningitidis*?**	5-7 days
***H. influenzae*?**	7-10 days
**Group B *streptococcus?***	14-21 days
**Gram negative bacilli?**	21 days

Ref: (1,7)

25) **How long would you expect fever to continue in uncomplicated bacterial meningitis?**
    Fever usually resolves within 5-7 days of therapy. Prolonged (>10d) fever occurs in 10%; usually caused by intercurrent viral infection, nosocomial or secondary bacterial infection, thrombophlebitis, or drug reaction.

Ref: (1)

26) **List complications of bacterial meningitis that may occur (CNS & non-CNS).**
    Acute CNS complications: seizures,increased ICP, cranial nerve palsies, stroke, cerebral or cerebellar herniation, thrombosis of dural venous sinuses, SIADH (syndrome of inappropriate antidiuretic hormone secretion), subdural effusions or empyema (10-30% of patients; asymptomatic in 85-90% of patients), intracranial abscess, ventriculitis

    Non-CNS complications: pericarditis, arthritis; especially occur with treatment for *N. meningitidis* meningitis, resulting from either bacterial dissemination or from immune complex deposition

    Ref: (1)
27) **What is the mortality rate of bacterial meningitis despite appropriate antibiotic therapy and supportive care?**

<10%; higher rates seen with *S. pneumoniae*

Ref: (1)

Oscar defervesces after 4 days, completes his course of antibiotics intravenously, and returns to his baseline self. Oscar’s parents wonder what to look out for following this episode of meningitis.

28) **Of those who survive, what percentage have some sort of morbidity? What are the most common neurologic sequelae following meningitis?**
    50% morbidity (but some subtle), with 10-20% of survivors having severe neurodevelopmental sequelae.
    Most common neurologic sequelae:

- Hearing loss (sensorineural hearing loss is MOST common and usually already present at time of initial presentation)
- Cognitive impairment
- Recurrent seizures
- Language development delay
- Visual impairment
- Behavioural problems

Ref: (1)

29) **List indicators of poor prognosis.**

- Age <6 months
- High concentration of bacteria in CSF
- Seizures occurring >4 days after the initiation of therapy
- Coma or focal neurological signs on presentation

Ref: (1)

30) **What is the mechanism of sensorineural hearing loss? How common is it? When should audiology testing be performed?**
    Direct cochlear infection OR direct inflammation of the auditory nerve.^1^ Occurs in 30% of pneumococcal meningitis, 5-20% of *H. influenzae* type b meningitis, 10% of meningococcal meningitis.^1^ Before discharge or shortly after discharge for ALL children diagnosed with meningitis (i.e., as soon as possible because cochlea can ossify quickly, compromising cochlear implant eligibility).^5^ If deficit detected, frequent follow-up and assessment for cochlear implants. If no deficit is detected, no follow-up audiology required.

Oscar is discharged home in stable condition with follow-up scheduled with his primary care provider next week. His family thanks you for your care.

***Did you know?***

Oscar Wilde, the prolific 19^th^ century writer best known for his play *The Importance of Being Earnest* and novel *The Picture of Dorian Gray*, died of meningitis, presumably following a chronic ear infection.

Learning Objectives

By the end of this session, participants should be able to:

1.	Apply an evidence-informed approach to the evaluation of infants and children with fever and altered mental status when bacterial meningitis is suspected.

2.	Recognize the epidemiologic, microbiologic, and clinical features that should raise suspicion for bacterial meningitis in infants and children.

3.	Identify indications and contraindications for lumbar puncture and interpret the effect of prior antibiotic therapy on the diagnostic yield of cerebrospinal fluid and blood cultures.

4.	Select appropriate empiric antimicrobial therapy and adjunctive corticosteroid therapy for suspected bacterial meningitis and determine pathogen-directed treatment duration.

5.	Recommend appropriate follow-up after bacterial meningitis, including evaluation for important long-term complications such as hearing loss.

Suggested Readings:

Janowski AB, Hunstad DA. Central Nervous System Infections. In: Kliegman RM, St Geme JW, Blum NJ, Shah SS, Tasker RC, Wilson KM, editors. **Nelson Textbook of Pediatrics.** 21st ed. Philadelphia, PA: Elsevier Inc.; 2020. p. 3222–34.

**CPS Statement:** Le Saux N. Guidelines for the management of suspected and confirmed bacterial meningitis in Canadian children older than 2 months of age [Internet]. Canadian Paediatric Society Position Statement. 2020. Available from: <https://www.cps.ca/en/documents/position/management-of-bacterial-meningitis>

Reference List:

1. Janowski AB, Hunstad DA. Central Nervous System Infections. In: Kliegman RM, St Geme JW, Blum NJ, Shah SS, Tasker RC, Wilson KM, editors. Nelson Textbook of Pediatrics. 21st ed. Philadelphia, PA: Elsevier Inc.; 2020. p. 3222–34.
2. Amarilyo G, Alper A, Ben-Tov A, Grisaru-Soen G. Diagnostic Accuracy of Clinical Symptoms and Signs in Children With Meningitis. Pediatr Emerg Care [Internet]. 2011;27(3):196–9. Available from: http://content.wkhealth.com/linkback/openurl?sid=WKPTLP:landingpage&an=00006565-201103000-00008
3. Thomas KE, Hasbun R, Jekel J, Quagliarello VJ. The diagnostic accuracy of Kernig’s sign, Brudzinski’s sign, and nuchal rigidity in adults with suspected meningitis. Clin Infect Dis. 2002;35(June 1999):46–52.
4. Hartman ME, Cheifetz IM. Pediatric Emergencies and Resuscitation. In: Kliegman RM, St Geme JW, Blum NJ, Shah SS, Tasker RC, Wilson KM, editors. Nelson Textbook of Pediatrics. 21st ed. Philadelphia, PA: Elsevier Inc.; 2020. p. 530–47.
5. Hasbun R, Van de Beek D, Brouwer MC, Tunkel AR. Acute Meningitis. In: Bennett JE, Dolin R, Blaser MJ, editors. Mandell, Douglas, and Bennett’s Principles and Practice of Infectious DIseases. 9th ed. Philadelphia, PA: Elsevier Inc.; 2020. p. 1183–219.
6. Morgan A Alford, James A Karlowsky, Heather J Adam, Melanie R Baxter, John Schellenberg, Alyssa R Golden, Irene Martin, Walter Demczuk, Michael R Mulvey, George G Zhanel, on behalf of the Canadian Antimicrobial Resistance Alliance (CARA), Antimicrobial susceptibility testing of invasive isolates of *Streptococcus pneumoniae* from Canadian patients: the SAVE study, 2011–2020, *Journal of Antimicrobial Chemotherapy*, Volume 78, Issue Supplement_1, May 2023, Pages i8–i16, <https://doi.org/10.1093/jac/dkad065>
7. Le Saux N. Guidelines for the management of suspected and confirmed bacterial meningitis in Canadian children older than 2 months of age [Internet]. Canadian Paediatric Society Position Statement. 2020 [cited 2021 Jul 2]. Available from: https://www.cps.ca/en/documents/position/management-of-bacterial-meningitis
8. van de Beek, D., de Gans, J., McIntyre, P., & Prasad, K. (2004). Steroids in adults with acute bacterial meningitis: a systematic review. *The Lancet Infectious Diseases*, *4*(3), 139–143. https://doi.org/10.1016/S1473-3099(04)00937-5
9. Gundamraj S, Hasbun R. The Use of Adjunctive Steroids in Central Nervous Infections. Front Cell Infect Microbiol. 2020 Nov 23;10:592017. doi: 10.3389/fcimb.2020.592017. PMID: 33330135; PMCID: PMC7719626.
10. Mohanty S, Feemster K, Yu KC, Watts JA, Gupta V. Trends in *Streptococcus pneumoniae* Antimicrobial Resistance in US Children: A Multicenter Evaluation. Open Forum Infect Dis. 2023 Mar 7;10(3):ofad098. doi: 10.1093/ofid/ofad098. PMID: 36968964; PMCID: PMC10034583.
11. Allan R. Tunkel, Barry J. Hartman, Sheldon L. Kaplan, Bruce A. Kaufman, Karen L. Roos, W. Michael Scheld, Richard J. Whitley, Practice Guidelines for the Management of Bacterial Meningitis, Clinical Infectious Diseases, Volume 39, Issue 9, 1 November 2004, Pages 1267–1284, <https://doi.org/10.1086/425368>
12. Lin, W. L., Chi, H., Huang, F. Y., Huang, D. T., & Chiu, N. C. (2016). Analysis of clinical outcomes in pediatric bacterial meningitis focusing on patients without cerebrospinal fluid pleocytosis. *Journal of microbiology, immunology, and infection = Wei mian yu gan ran za zhi*, *49*(5), 723–728. <https://doi.org/10.1016/j.jmii.2014.08.013>
13. Greenberg RG, Smith PB, Cotten CM, Moody MA, Clark RH, Benjamin DK Jr. Traumatic lumbar punctures in neonates: test performance of the cerebrospinal fluid white blood cell count. *Pediatr Infect Dis J.* 2008;27(12):1047-1051. doi:10.1097/INF.0b013e31817e519b
14. Kanegaye JT, Soliemanzadeh P, Bradley JS. Lumbar puncture in pediatric bacterial meningitis: defining the time interval for recovery of cerebrospinal fluid pathogens after parenteral antibiotic pretreatment. *Pediatrics.* 2001;108(5):1169-1174.
15. Michael B, Menezes BF, Cunniffe J, Miller A, Kneen R, Francis G, Beeching NJ, Solomon T. Effect of delayed lumbar punctures on the diagnosis of acute bacterial meningitis in adults. *Emerg Med J.* 2010;27(6):433-438. doi:10.1136/emj.2009.075598
16. Cherry JD, Kaplan SL, Demmler-Harrison GJ, Steinbach WJ, Hotez PJ, Williams JV, eds. *Feigin and Cherry’s Textbook of Pediatric Infectious Diseases*. 9th ed. Elsevier; 2024.
17. Adriani KS, Brouwer MC, van de Beek D. Risk factors for community-acquired bacterial meningitis in adults. *Neth J Med*. 2015;73(2):53-60.
18. Gounder PP, Zulz T, Desai S, et al. Epidemiology of bacterial meningitis in the North American Arctic, 2000-2010. *J Infect*. 2015;71(2):179-187.
19. Reefhuis J, Honein MA, Whitney CG, et al. Risk of bacterial meningitis in children with cochlear implants. *N Engl J Med*. 2003;349(5):435-445. doi:10.1056/NEJMoa031101
