# Supplementary material for: From Presentation to Prognosis: Case-Based Learning in Pediatric Bacterial Meningitis for Postgraduate Training
Source: MedEdPORTAL. 2026 Aug 25;22:11632. doi: 10.15766/mep_2374-8265.11632 (PMC13503623; doi:10.15766/mep_2374-8265.11632)
Supplement: Supplementary file 1 [file mep_2374-8265.11632-s001.zip › B. Module - Student Version.docx]

**An Infant with Fever and Altered Mental Status**

Case-based learning session

**The Case:**
Oscar is an 8-month-old boy whom you are seeing in the Emergency Department. His mother reports that he has had a runny nose and fever for the past few days. She gave him acetaminophen and a dose of amoxicillin that she had left over from an illness one month prior, which seemed to help his fever. Today, however, she noticed that he was not interested in eating or drinking and was acting very sleepy. Worried, she brought him to the hospital.

**Past Medical History:**

- Born at term via spontaneous vaginal delivery, no perinatal or neonatal complications
- No previous hospitalizations or surgeries
- Treated for an ear infection one month ago
- Immunizations are up to date for age, as per the local immunization schedule for his age with adequate doses of pneumococcal conjugate vaccine for age

**Social History:**

- Attends day care 5 days a week
- Lives with his parents and two siblings (4-year-old who attends daycare; 7-year-old is in Grade 1)

**On Physical Exam:**

- General appearance:	Lethargic and ill-looking with mottled skin, capillary refill 3-4 seconds
- Vitals:	T 39.7°C, HR 156 bpm, RR 37 breaths/min, BP 85/48 mmHg, SpO2 98%
- Weight:	8.9 kg
- HEENT:	Erythematous left tympanic membrane, normal positioned pinnae, no tenderness to palpation over the mastoids
- NEURO:	Irritable cry in response to pain, eye opening to verbal stimuli, limbs withdraw to touch, flat anterior fontanelle, pupils equal and reactive to light, possible increased crying on passive flexion of the neck, Kernig and Brudzinski signs negative
- Chest:	Clear, no increased work of breathing
- CVS:	Sinus tachycardia, normal heart sounds S1 & S2, no murmur
- Abdomen:	Soft, non-tender, bowel sounds present

(1) **Summarize the pertinent points of the clinical case so far.**
(2) **What is the differential diagnosis you should consider?**
(3) **What are the Kernig’s and Brudzinski’s signs? How sensitive are these signs in children?**
(4) **What is Oscar’s Glasgow Coma Scale score?**
(5) **Does Oscar have evidence of increased intracranial pressure (ICP)? Why or why not?**
(6) **What further information on history and/or physical exam would be helpful in your assessment?**
(7) **What initial management and work-up would you do?**
(8) **A medical student asks you: “How useful is doing a blood culture when you are clinically suspecting meningitis?” How do you reply?**

You are planning to do a lumbar puncture (LP) as part of your initial work-up.

(9) **Name 3 contraindications to consider before doing your LP.**
(10) **If you suspect increased ICP, what should be done prior to attempting an LP?**
(11) **Suppose performing an LP was delayed by several days due to contraindications, but now, those are no longer present, and an LP could be performed. What useful information could an LP provide despite the patient already having received antibiotics for a few days? What additional test you may consider in the CSF to increase the yield of knowing the causative organism?**

(12) **How often is the CSF culture positive in community acquired bacterial meningitis if no previous antibiotics have been given? What about if antibiotics have been started?**

You would like to begin antibiotics even before test results return because meningitis and sepsis are on your differential and you do not want to delay treatment.

(13) **What organisms do you want to cover?**

(14) **Which antibiotic(s) would you start and why?**

(15) **What if the patient was allergic to B-lactam antibiotics?**
(16) **Would you start adjunctive dexamethasone? Why or why not?**

The following are select preliminary lab results:

**CBC**:	Hgb 154 g/L, RBC 5 x 10^12^/L, Platelets 297 x 10^9^/L, WBC 21 x 10^9^/L, neutrophils 40%, bands 24%, lymphocytes 34%

**Renal function & liver enzymes**: Normal

**Urinalysis**: Normal

**Urine culture:** Pending

**Blood culture:** Pending

**CSF**: Xanthochromic, WBC 210 x 10^6^/L (70% neutrophils), RBC 2820 x10^6^/L, glucose 0.2 mmol/L, protein 2.8 g/L; culture pending

(17) **Fill in the following table ++/ +/ normal / - / -- describe typical cell count and chemistry from CSF in the various infectious meningitic conditions:**

| **Component** | **Abnormal values** | | |
| --- | --- | --- | --- |
|  | **Bacterial** | **Viral** | **TB** |
| **WBC** |  |  |  |
| **% neutrophils** |  |  |  |
| **Protein** |  |  |  |
| **Glucose** |  |  |  |

(18) **How would you interpret the cell count of a traumatic LP?**

Oscar’s CSF Gram stain shows Gram positive cocci in pairs:

Centers for Disease Control and Prevention (CDC). Image ID 18081 [image]. Public domain. Atlanta (GA): CDC; [cited 2026 Feb 4]. Available from: <https://wwwn.cdc.gov/phil/Details.aspx?pid=18081>

(19) **What is your working diagnosis now?**

(20) **List risk factors for bacterial meningitis.**
(21) **What is the likely mode of transmission in meningitis?**
(22) **How do bacteria get to the meninges? What was the most likely route in Oscar?**
(23) **List signs and symptoms of meningitis in infants and in older children.**

| Infants | Older Children |
| --- | --- |
|  |  |

After 48 hours of intravenous antibiotics treatment, Oscar’s status is improving. He is more interactive with his parents and his fever trend is improving. His CSF culture was negative, but his blood culture grew *S. pneumoniae*, which was **intermediately susceptible to penicillin** (MIC 0.06-0.12 mcg/mL) and **susceptible to cefotaxime** according to CNS breakpoints.

(24) **What antibiotic would you use to treat Oscar? How long do you anticipate the duration of treatment would be (assuming he does not develop any complications)?**

**Assuming uncomplicated meningitis, what duration would you use if the causative organism had been each of the following:
*N. meningitidis*?**
***H. influenzae*?**
**Group B *streptococcus?***
**Gram negative bacilli?**

(25) **How long would you expect fever to continue in uncomplicated bacterial meningitis?**

(26) **List complications of bacterial meningitis that may occur (CNS & non-CNS).**
(27) **What is the mortality rate of bacterial meningitis despite appropriate antibiotic therapy and supportive care?**

Oscar defervesces after 4 days, completes his course of antibiotics intravenously, and returns to his baseline self. Oscar’s parents wonder what to look out for following this episode of meningitis.

(28) **Of those who survive, what percentage have some sort of morbidity? What are the most common neurologic sequelae following meningitis?**
(29) **List indicators of poor prognosis.**

(30) **What is the mechanism of the sensorineural hearing loss? How common is it? When should audiology testing be performed?**

Oscar is discharged home in stable condition with follow-up scheduled with his primary care provider next week. His family thanks you for your care.

***Did you know?***

Oscar Wilde, the prolific 19^th^ century writer best known for his play *The Importance of Being Earnest* and novel *The Picture of Dorian Gray*, died of meningitis, presumably following a chronic ear infection.

Learning Objectives

By the end of this session, participants should be able to:

1.	Apply an evidence-informed approach to the evaluation of infants and children with fever and altered mental status when bacterial meningitis is suspected.

2.	Recognize the epidemiologic, microbiologic, and clinical features that should raise suspicion for bacterial meningitis in infants and children.

3.	Identify indications and contraindications for lumbar puncture and interpret the effect of prior antibiotic therapy on the diagnostic yield of cerebrospinal fluid and blood cultures.

4.	Select appropriate empiric antimicrobial therapy and adjunctive corticosteroid therapy for suspected bacterial meningitis and determine pathogen-directed treatment duration.

5.	Recommend appropriate follow-up after bacterial meningitis, including evaluation for important long-term complications such as hearing loss.

Suggested Readings:

Janowski AB, Hunstad DA. Central Nervous System Infections. In: Kliegman RM, St Geme JW, Blum NJ, Shah SS, Tasker RC, Wilson KM, editors. **Nelson Textbook of Pediatrics.** 21st ed. Philadelphia, PA: Elsevier Inc.; 2020. p. 3222–34.

**CPS Statement:** Le Saux N. Guidelines for the management of suspected and confirmed bacterial meningitis in Canadian children older than 2 months of age [Internet]. Canadian Paediatric Society Position Statement. 2020. Available from: <https://www.cps.ca/en/documents/position/management-of-bacterial-meningitis>

Reference List:

1. Janowski AB, Hunstad DA. Central Nervous System Infections. In: Kliegman RM, St Geme JW, Blum NJ, Shah SS, Tasker RC, Wilson KM, editors. Nelson Textbook of Pediatrics. 21st ed. Philadelphia, PA: Elsevier Inc.; 2020. p. 3222–34.
2. Amarilyo G, Alper A, Ben-Tov A, Grisaru-Soen G. Diagnostic Accuracy of Clinical Symptoms and Signs in Children With Meningitis. Pediatr Emerg Care [Internet]. 2011;27(3):196–9. Available from: http://content.wkhealth.com/linkback/openurl?sid=WKPTLP:landingpage&an=00006565-201103000-00008
3. Thomas KE, Hasbun R, Jekel J, Quagliarello VJ. The diagnostic accuracy of Kernig’s sign, Brudzinski’s sign, and nuchal rigidity in adults with suspected meningitis. Clin Infect Dis. 2002;35(June 1999):46–52.
4. Hartman ME, Cheifetz IM. Pediatric Emergencies and Resuscitation. In: Kliegman RM, St Geme JW, Blum NJ, Shah SS, Tasker RC, Wilson KM, editors. Nelson Textbook of Pediatrics. 21st ed. Philadelphia, PA: Elsevier Inc.; 2020. p. 530–47.
5. Hasbun R, Van de Beek D, Brouwer MC, Tunkel AR. Acute Meningitis. In: Bennett JE, Dolin R, Blaser MJ, editors. Mandell, Douglas, and Bennett’s Principles and Practice of Infectious DIseases. 9th ed. Philadelphia, PA: Elsevier Inc.; 2020. p. 1183–219.
6. Morgan A Alford, James A Karlowsky, Heather J Adam, Melanie R Baxter, John Schellenberg, Alyssa R Golden, Irene Martin, Walter Demczuk, Michael R Mulvey, George G Zhanel, on behalf of the Canadian Antimicrobial Resistance Alliance (CARA), Antimicrobial susceptibility testing of invasive isolates of *Streptococcus pneumoniae* from Canadian patients: the SAVE study, 2011–2020, *Journal of Antimicrobial Chemotherapy*, Volume 78, Issue Supplement_1, May 2023, Pages i8–i16, <https://doi.org/10.1093/jac/dkad065>
7. Le Saux N. Guidelines for the management of suspected and confirmed bacterial meningitis in Canadian children older than 2 months of age [Internet]. Canadian Paediatric Society Position Statement. 2020 [cited 2021 Jul 2]. Available from: https://www.cps.ca/en/documents/position/management-of-bacterial-meningitis
8. van de Beek, D., de Gans, J., McIntyre, P., & Prasad, K. (2004). Steroids in adults with acute bacterial meningitis: a systematic review. *The Lancet Infectious Diseases*, *4*(3), 139–143. https://doi.org/10.1016/S1473-3099(04)00937-5
9. Gundamraj S, Hasbun R. The Use of Adjunctive Steroids in Central Nervous Infections. Front Cell Infect Microbiol. 2020 Nov 23;10:592017. doi: 10.3389/fcimb.2020.592017. PMID: 33330135; PMCID: PMC7719626.
10. Mohanty S, Feemster K, Yu KC, Watts JA, Gupta V. Trends in *Streptococcus pneumoniae* Antimicrobial Resistance in US Children: A Multicenter Evaluation. Open Forum Infect Dis. 2023 Mar 7;10(3):ofad098. doi: 10.1093/ofid/ofad098. PMID: 36968964; PMCID: PMC10034583.
11. Allan R. Tunkel, Barry J. Hartman, Sheldon L. Kaplan, Bruce A. Kaufman, Karen L. Roos, W. Michael Scheld, Richard J. Whitley, Practice Guidelines for the Management of Bacterial Meningitis, Clinical Infectious Diseases, Volume 39, Issue 9, 1 November 2004, Pages 1267–1284, <https://doi.org/10.1086/425368>
12. Lin, W. L., Chi, H., Huang, F. Y., Huang, D. T., & Chiu, N. C. (2016). Analysis of clinical outcomes in pediatric bacterial meningitis focusing on patients without cerebrospinal fluid pleocytosis. *Journal of microbiology, immunology, and infection = Wei mian yu gan ran za zhi*, *49*(5), 723–728. <https://doi.org/10.1016/j.jmii.2014.08.013>
13. Greenberg RG, Smith PB, Cotten CM, Moody MA, Clark RH, Benjamin DK Jr. Traumatic lumbar punctures in neonates: test performance of the cerebrospinal fluid white blood cell count. *Pediatr Infect Dis J.* 2008;27(12):1047-1051. doi:10.1097/INF.0b013e31817e519b
14. Kanegaye JT, Soliemanzadeh P, Bradley JS. Lumbar puncture in pediatric bacterial meningitis: defining the time interval for recovery of cerebrospinal fluid pathogens after parenteral antibiotic pretreatment. *Pediatrics.* 2001;108(5):1169-1174.
15. Michael B, Menezes BF, Cunniffe J, Miller A, Kneen R, Francis G, Beeching NJ, Solomon T. Effect of delayed lumbar punctures on the diagnosis of acute bacterial meningitis in adults. *Emerg Med J.* 2010;27(6):433-438. doi:10.1136/emj.2009.075598
16. Cherry JD, Kaplan SL, Demmler-Harrison GJ, Steinbach WJ, Hotez PJ, Williams JV, eds. *Feigin and Cherry’s Textbook of Pediatric Infectious Diseases*. 9th ed. Elsevier; 2024.
17. Adriani KS, Brouwer MC, van de Beek D. Risk factors for community-acquired bacterial meningitis in adults. *Neth J Med*. 2015;73(2):53-60.
18. Gounder PP, Zulz T, Desai S, et al. Epidemiology of bacterial meningitis in the North American Arctic, 2000-2010. *J Infect*. 2015;71(2):179-187.
19. Reefhuis J, Honein MA, Whitney CG, et al. Risk of bacterial meningitis in children with cochlear implants. *N Engl J Med*. 2003;349(5):435-445. doi:10.1056/NEJMoa031101
