# Supplementary material for: From Presentation to Prognosis: Case-Based Learning in Pediatric Bacterial Meningitis for Postgraduate Training
Source: MedEdPORTAL. 2026 Aug 25;22:11632. doi: 10.15766/mep_2374-8265.11632 (PMC13503623; doi:10.15766/mep_2374-8265.11632)
Supplement: Supplementary file 1 [file mep_2374-8265.11632-s001.zip › D. Trainee Feedback Form.docx]

**Meningitis Case Based Learning – Trainee Feedback Form**

*Post-session Evaluation Form (Trainee)*

By the end of this session, learners will be able to:

1.	Apply an evidence-informed approach to the evaluation of infants and children with fever and altered mental status when bacterial meningitis is suspected.

2.	Recognize the epidemiologic, microbiologic, and clinical features that should raise suspicion for bacterial meningitis in infants and children.

3.	Identify indications and contraindications for lumbar puncture and interpret the effect of prior antibiotic therapy on the diagnostic yield of cerebrospinal fluid and blood cultures.

4.	Select appropriate empiric antimicrobial therapy and adjunctive corticosteroid therapy for suspected bacterial meningitis and determine pathogen-directed treatment duration.

5.	Recommend appropriate follow-up after bacterial meningitis, including evaluation for important long-term complications such as hearing loss.

**Site/hospital:**	_________________________________

**Date:**	_________________________________

**Level of training:**	_________________________________

**Facilitator’s name:**	_________________________________

**Did you review the pre-session materials to prepare for this session? (circle)**

Yes / No

**If yes, how much time did you spend preparing (minutes)?**

________________________

**What did you review in preparation for this session? (check all that apply)**

- Skimmed the case
- Reviewed one reference article
- Reviewed more than one reference article
- Other: ________________________

**Please check off the following items on a scale of 1–5:**

|  | Not applicable | 1 = Strongly disagree | 2 | 3 | 4 | 5 = Strongly agree |
| --- | --- | --- | --- | --- | --- | --- |
| **1. Pre-session materials were useful in preparing for the session.** |  |  |  |  |  |  |
| **2. The facilitator encouraged participation of all group members.** |  |  |  |  |  |  |
| **3. The facilitator was well prepared for the session.** |  |  |  |  |  |  |
| **4. The small group discussion was engaging.** |  |  |  |  |  |  |
| **5. The case-based format promoted active learning.** |  |  |  |  |  |  |
| **6. I achieved the learning objectives listed above.** |  |  |  |  |  |  |

**Global assessment – how would you rate this session? (check off)**

| 1 = very poor | 2 | 3 | 4 | 5 = one of the best sessions for learning in the curriculum |
| --- | --- | --- | --- | --- |
|  |  |  |  |  |

**Was this module a realistic case?** **(circle)**	Yes / No

**Was this module challenging enough? (circle)**	Yes / No

**What did you learn from the session that was new to you?**

**Did this teaching session change your practice? (circle)**	Yes / No

**Would you participate in this type of session again? (circle)**	Yes / No

**Any other comments?**

***Thank you for your feedback!***
