# Supplementary material for: From Presentation to Prognosis: Case-Based Learning in Pediatric Bacterial Meningitis for Postgraduate Training
Source: MedEdPORTAL. 2026 Aug 25;22:11632. doi: 10.15766/mep_2374-8265.11632 (PMC13503623; doi:10.15766/mep_2374-8265.11632)
Supplement: Supplementary file 1 [file mep_2374-8265.11632-s001.zip › E. Facilitator Feedback Form.docx]

**Meningitis Case Based Learning – Facilitator Feedback Form**

*Post-session Evaluation Form (Facilitator)*

By the end of this session, learners will be able to:

1.	Apply an evidence-informed approach to the evaluation of infants and children with fever and altered mental status when bacterial meningitis is suspected.

2.	Recognize the epidemiologic, microbiologic, and clinical features that should raise suspicion for bacterial meningitis in infants and children.

3.	Identify indications and contraindications for lumbar puncture and interpret the effect of prior antibiotic therapy on the diagnostic yield of cerebrospinal fluid and blood cultures.

4.	Select appropriate empiric antimicrobial therapy and adjunctive corticosteroid therapy for suspected bacterial meningitis and determine pathogen-directed treatment duration.

5.	Recommend appropriate follow-up after bacterial meningitis, including evaluation for important long-term complications such as hearing loss.

**Site/hospital:**	_________________________________

**Date:**	_________________________________

**Your level of training (e.g. attending physician, fellow, etc.):** _______________________________

**Level of training of your learner audience (select all that apply):**

- Medical student
- PGY-1
- PGY-2
- PGY-3
- PGY-4
- Infectious Diseases fellow
- Infectious Diseases attending
- Other: ________________________

**Total number of learners in your group:** _____________

**Delivery mode (select all that apply):**

- In person
- Online/Virtual
- Hybrid

**What did you do to prepare for this session (select all that apply)?**

- Read over answer key
- Read additional articles
- Reviewed personal notes on this topic
- Reviewed previous talks on this topic
- Other: ________________________

**Time spent preparing to be a facilitator for this session (minutes):** _______________________

**What did you review in preparation for this session?**

- Skimmed the case
- Reviewed one reference article
- Reviewed more than one reference article
- Other: ________________________

**Time you would normally have spent to prepare for a teaching session on this topic if you had to prepare your own teaching materials (minutes):** ________________________

**Please provide comments on the pre-circulated materials (case, answer key, and articles). Were they useful for your preparation? Do you have any suggestions for improving the case, answer key, or article list?**

**Please provide comments on the case-based learning format from your observations of the trainees. Did it promote clinical reasoning and/or critical thinking?**

**Would you be willing to be a facilitator for this type of session again? (circle)** Yes / No

**Any other comments?**

***Thank you for your feedback!***
