# Supplementary material for: From Presentation to Prognosis: Case-Based Learning in Pediatric Bacterial Meningitis for Postgraduate Training
Source: MedEdPORTAL. 2026 Aug 25;22:11632. doi: 10.15766/mep_2374-8265.11632 (PMC13503623; doi:10.15766/mep_2374-8265.11632)
Supplement: Supplementary file 1 [file mep_2374-8265.11632-s001.zip › G. Pre and Posttest - Answers.docx]

PRE AND POST-TEST ANSWERS

1. What is the main role of adding vancomycin in cases of suspected meningitis?
   a. To ensure therapeutic antibiotic levels in the CNS since it is amenable to therapeutic drug monitoring
   b. To cover for possible MRSA (methicillin-resistant *Staphylococcus aureus)*
   c. **To cover for possible resistant *Streptococcus pneumoniae***
   d. To cover for coagulase-negative staphylococci
2. The main rationale of using corticosteroids in meningitis is:
   a. To increase antibiotic penetration to the brain
   b. **To decrease hearing loss following infection**
   c. To help with the systemic inflammatory response
   d. There is no rationale to using corticosteroids in meningitis
3. Which of the following is NOT a contraindication to doing a lumbar puncture?
   a. Clear signs of increased intracranial pressure
   b. Skin infection overlying the site of the LP
   c. Coagulopathy
   d. **Positive Kernig’s or Brudzinski’s sign**
4. Approximately how often is the CSF culture positive when a lumbar puncture is performed after 6 hours of effective antibiotic therapy?
   a. 80-90% of the time
   b. **1-50% the time**
   c. Less than 1% of the time
   d. Never
5. How often is the blood culture positive in meningitis if antibiotics have not yet been given?
   a. **80-90% of the time**
   b. Less than half the time
   c. Less than 1% of the time
   d. Never
6. What is the recommended antibiotic duration for uncomplicated *Streptococcus pneumoniae* meningitis (uncomplicated)?
   a. 5-7 days
   b. 7-10 days
   c. **10-14 days**
   d. 14-21 days
7. What test should be performed in ALL children with meningitis, ideally prior to discharge?
   a. Repeat lumbar puncture
   b. **Hearing test**
   c. MRI brain
   d. Eye exam
8. Name one thing that you learned from this module that you didn’t know before:
